# *N*,*N*′-Bis(3,5-dichloro­benzyl­idene)­ethane-1,2-diamine

**DOI:** 10.1107/S1600536808033588

**Published:** 2008-10-18

**Authors:** Hoong-Kun Fun, Reza Kia

**Affiliations:** a X-ray Crystallography Unit, School of Physics, Universiti Sains Malaysia, 11800 USM, Penang, Malaysia

## Abstract

The mol­ecule of the title Schiff base compound, C_16_H_12_Cl_4_N_2_, lies across an inversion centre and adopts an *E* configuration with respect to the azomethine C=N bond. The imine groups are coplanar with the aromatic rings. Within the mol­ecule, the planar units are parallel but extend in opposite directions from the dimethyl­ene bridge. In the crystal structure, mol­ecules are linked together by inter­molecular C—H⋯Cl hydrogen bonds along the *a* axis.

## Related literature

For bond-length data, see: Allen *et al.* (1987 ▶). For related structures, see, for example: Fun & Kia (2008*a* ▶,*b* ▶,*c* ▶); Fun, Kargar & Kia (2008 ▶); Fun, Kia & Kargar (2008 ▶). For information on Schiff base complexes and their applications, see, for example: Pal *et al.* (2005 ▶); Calligaris & Randaccio (1987 ▶); Hou *et al.* (2001 ▶); Ren *et al.* (2002 ▶).
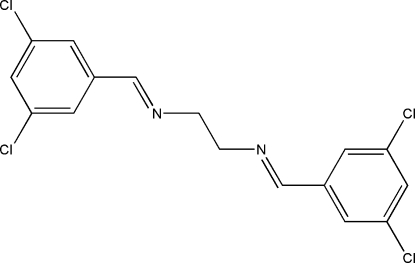


## Experimental

### 

#### Crystal data

C_16_H_12_Cl_4_N_2_
*M* *_r_* = 374.08
Monoclinic, 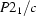

*a* = 8.0539 (3) Å
*b* = 14.0170 (4) Å
*c* = 7.5015 (3) Å
β = 110.612 (1)°
*V* = 792.64 (5) Å^3^
*Z* = 2
Mo *K*α radiation
μ = 0.74 mm^−1^
*T* = 100.0 (1) K
0.52 × 0.25 × 0.13 mm

#### Data collection

Bruker SMART APEXII CCD area-detector diffractometer
Absorption correction: multi-scan (**SADABS**; Bruker, 2005 ▶) *T*_min_ = 0.699, *T*_max_ = 0.908
34536 measured reflections
4162 independent reflections
3485 reflections with *I* > 2σ(*I*)
*R*_int_ = 0.035

#### Refinement

*R*[*F* ^2^ > 2σ(*F* ^2^)] = 0.034
*wR*(*F* ^2^) = 0.097
*S* = 1.06
4162 reflections
124 parameters
H atoms treated by a mixture of independent and constrained refinement
Δρ_max_ = 0.70 e Å^−3^
Δρ_min_ = −0.25 e Å^−3^

### 

Data collection: *APEX2* (Bruker, 2005 ▶); cell refinement: *SAINT* (Bruker, 2005 ▶); data reduction: *SAINT*; program(s) used to solve structure: *SHELXTL* (Sheldrick, 2008 ▶); program(s) used to refine structure: *SHELXTL*; molecular graphics: *SHELXTL*; software used to prepare material for publication: *SHELXTL* and *PLATON* (Spek, 2003 ▶).

## Supplementary Material

Crystal structure: contains datablocks global, I. DOI: 10.1107/S1600536808033588/bg2206sup1.cif

Structure factors: contains datablocks I. DOI: 10.1107/S1600536808033588/bg2206Isup2.hkl

Additional supplementary materials:  crystallographic information; 3D view; checkCIF report

## Figures and Tables

**Table 1:** Hydrogen-bond geometry (Å, °)

*D*—H⋯*A*	*D*—H	H⋯*A*	*D*⋯*A*	*D*—H⋯*A*
C1—H1⋯Cl2^i^	0.962 (14)	2.830 (16)	3.6479 (9)	143.5 (13)

Symmetry code: (i) 
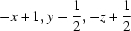
.

**Fig. 1.**
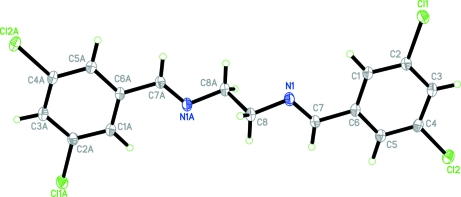
The molecular structure of (I) with atom labels and 50% probability ellipsoids for non-H atoms. The suffix A corresponds to symmetry code (-x + 2, -y, -z + 1).

**Fig. 2.**
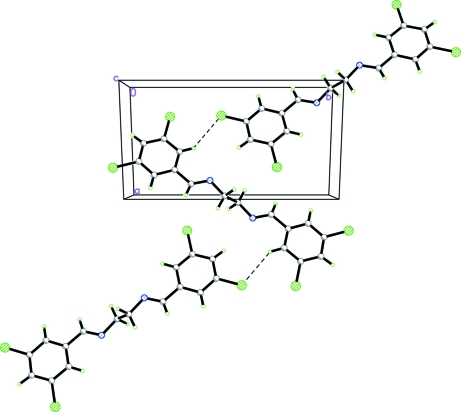
The crystal packing of (I), viewed down the c-axis, showing the linking of the molecules by intermolecular C—H···Cl hydrogen bonds along the a-axis. Intermolecular hydrogen bonds are shown as dashed lines.

## supplementary crystallographic information

### Comment

Schiff bases are among the most prevalent mixed-donor ligands in the field of coordination chemistry in which there has been growing interest, mainly because of their wide applications in areas such as biochemistry, synthesis, and catalysis (Pal *et al.*, 2005; Hou *et al.*, 2001; Ren *et al.*, 2002). Many Schiff base complexes have been structurally characterized, but only a relatively small number of free Schiff bases have had their X-ray structures reported (Calligaris & Randaccio, 1987). As an extension of our work (Fun, Kargar & Kia 2008; Fun, Kia & Kargar 2008) on the structural characterization of Schiff base ligands, the title compound (I), is reported here.

The molecule of the title compound (Fig. 1), lies across an inversion centre and adopts an *E* configuration with respect to the azomethine C═N bond. The bond lengths and angles are within normal ranges (Allen *et al.*, 1987) and are comparable with the values found in related structures (Fun & Kia (2008*a*,*b*,*c*); Fun, Kargar & Kia 2008; Fun, Kia & Kargar 2008). The two planar units are parallel but extend in opposite directions from the dimethylene bridge. In the crystal structure, molecules are linked together by intermolecular C—H···Cl hydrogen bonds along the *a*-axis.

### Experimental

The synthetic method has been described earlier (Fun, Kargar, & Kia, 2008). Single crystals suitable for *X*-ray diffraction were obtained by evaporation of an ethanol solution at room temperature.

### Refinement

All of the hydrogen atoms were located from the difference Fourier map and refined freely. The highest peak is located 0.63 Å from C7 and the deepest hole is located 0.55 Å from Cl2.

### Crystal data

**Table:**

C_16_H_12_Cl_4_N_2_	*F*(000) = 380
*M**_r_* = 374.08	*D*_x_ = 1.567 Mg m^−^^3^
Monoclinic, *P*2_1_/*c*	Mo *K*α radiation, λ = 0.71073 Å
Hall symbol: -P 2ybc	Cell parameters from 9889 reflections
*a* = 8.0539 (3) Å	θ = 2.7–39.9°
*b* = 14.0170 (4) Å	µ = 0.74 mm^−^^1^
*c* = 7.5015 (3) Å	*T* = 100 K
β = 110.612 (1)°	Block, colourless
*V* = 792.64 (5) Å^3^	0.52 × 0.25 × 0.13 mm
*Z* = 2	

### Data collection

**Table:**

Bruker SMART APEXII CCD area-detector diffractometer	4162 independent reflections
Radiation source: fine-focus sealed tube	3485 reflections with *I* > 2σ(*I*)
graphite	*R*_int_ = 0.035
CCD rotation images, thin slices scans	θ_max_ = 37.5°, θ_min_ = 2.7°
Absorption correction: multi-scan (*SADABS*; Bruker, 2005)	*h* = −13→13
*T*_min_ = 0.699, *T*_max_ = 0.908	*k* = −23→24
34536 measured reflections	*l* = −12→12

### Refinement

**Table:**

Refinement on *F*^2^	Primary atom site location: structure-invariant direct methods
Least-squares matrix: full	Secondary atom site location: refall
*R*[*F*^2^ > 2σ(*F*^2^)] = 0.034	Hydrogen site location: inferred from neighbouring sites
*wR*(*F*^2^) = 0.097	H atoms treated by a mixture of independent and constrained refinement
*S* = 1.06	*w* = 1/[σ^2^(*F*_o_^2^) + (0.0543*P*)^2^ + 0.1408*P*] where *P* = (*F*_o_^2^ + 2*F*_c_^2^)/3
4162 reflections	(Δ/σ)_max_ = 0.001
124 parameters	Δρ_max_ = 0.70 e Å^−^^3^
0 restraints	Δρ_min_ = −0.25 e Å^−^^3^

### Special details

**Table:**

Experimental. The low-temperature data was collected with the Oxford Cyrosystem Cobra low-temperature attachment.
Geometry. All esds (except the esd in the dihedral angle between two l.s. planes) are estimated using the full covariance matrix. The cell esds are taken into account individually in the estimation of esds in distances, angles and torsion angles; correlations between esds in cell parameters are only used when they are defined by crystal symmetry. An approximate (isotropic) treatment of cell esds is used for estimating esds involving l.s. planes.
Refinement. Refinement of F^2^ against ALL reflections. The weighted R-factor wR and goodness of fit S are based on F^2^, conventional R-factors R are based on F, with F set to zero for negative F^2^. The threshold expression of F^2^ > 2sigma(F^2^) is used only for calculating R-factors(gt) etc. and is not relevant to the choice of reflections for refinement. R-factors based on F^2^ are statistically about twice as large as those based on F, and R- factors based on ALL data will be even larger.

### Fractional atomic coordinates and isotropic or equivalent isotropic displacement parameters (Å^2^)

**Table:**

	*x*	*y*	*z*	*U*_iso_*/*U*_eq_	
Cl1	0.28013 (3)	0.291680 (16)	−0.02974 (3)	0.02218 (6)	
Cl2	0.72672 (3)	0.547370 (15)	0.42924 (4)	0.02557 (7)	
N1	0.83741 (10)	0.09945 (5)	0.45102 (12)	0.01963 (14)	
C1	0.58794 (11)	0.24294 (6)	0.24399 (13)	0.01689 (14)	
C2	0.47672 (11)	0.31735 (6)	0.15494 (13)	0.01727 (14)	
C3	0.51579 (12)	0.41204 (6)	0.20925 (13)	0.01900 (15)	
C4	0.67376 (12)	0.43026 (6)	0.35795 (13)	0.01858 (14)	
C5	0.78922 (11)	0.35774 (6)	0.45130 (13)	0.01811 (14)	
C6	0.74533 (11)	0.26360 (6)	0.39426 (12)	0.01621 (13)	
C7	0.86553 (11)	0.18627 (6)	0.49751 (12)	0.01690 (14)	
C8	0.96808 (13)	0.03109 (6)	0.56502 (14)	0.02076 (16)	
H1	0.558 (2)	0.1783 (10)	0.202 (2)	0.025 (4)*	
H3	0.447 (2)	0.4639 (12)	0.150 (2)	0.034 (4)*	
H5	0.8978 (18)	0.3723 (10)	0.5610 (19)	0.019 (3)*	
H7	0.956 (2)	0.2083 (11)	0.602 (2)	0.027 (4)*	
H8A	0.9095 (19)	−0.0120 (10)	0.641 (2)	0.019 (3)*	
H8B	1.071 (2)	0.0617 (11)	0.660 (2)	0.022 (3)*	

### Atomic displacement parameters (Å^2^)

**Table:**

	*U*^11^	*U*^22^	*U*^33^	*U*^12^	*U*^13^	*U*^23^
Cl1	0.01762 (9)	0.02168 (10)	0.02490 (11)	0.00313 (6)	0.00456 (8)	0.00146 (7)
Cl2	0.02981 (12)	0.01319 (9)	0.03115 (12)	0.00056 (7)	0.00756 (9)	−0.00046 (7)
N1	0.0189 (3)	0.0151 (3)	0.0234 (3)	0.0039 (2)	0.0056 (3)	0.0025 (2)
C1	0.0164 (3)	0.0142 (3)	0.0213 (3)	0.0015 (2)	0.0082 (3)	0.0015 (2)
C2	0.0160 (3)	0.0164 (3)	0.0203 (3)	0.0021 (2)	0.0075 (3)	0.0019 (3)
C3	0.0200 (3)	0.0151 (3)	0.0234 (4)	0.0038 (3)	0.0096 (3)	0.0030 (3)
C4	0.0211 (3)	0.0127 (3)	0.0234 (4)	0.0011 (3)	0.0098 (3)	0.0010 (3)
C5	0.0192 (3)	0.0148 (3)	0.0209 (3)	0.0013 (2)	0.0078 (3)	0.0011 (2)
C6	0.0167 (3)	0.0134 (3)	0.0200 (3)	0.0021 (2)	0.0083 (3)	0.0025 (2)
C7	0.0156 (3)	0.0160 (3)	0.0193 (3)	0.0031 (2)	0.0064 (3)	0.0018 (2)
C8	0.0218 (4)	0.0162 (3)	0.0227 (4)	0.0051 (3)	0.0058 (3)	0.0030 (3)

### Geometric parameters (Å, °)

**Table:**

Cl1—C2	1.7353 (9)	C3—H3	0.929 (17)
Cl2—C4	1.7326 (9)	C4—C5	1.3893 (12)
N1—C7	1.2643 (11)	C5—C6	1.3939 (12)
N1—C8	1.4571 (11)	C5—H5	0.988 (13)
C1—C2	1.3839 (11)	C6—C7	1.4778 (11)
C1—C6	1.3983 (12)	C7—H7	0.916 (16)
C1—H1	0.962 (14)	C8—C8^i^	1.526 (2)
C2—C3	1.3916 (12)	C8—H8A	1.050 (15)
C3—C4	1.3888 (13)	C8—H8B	0.979 (15)
			
C7—N1—C8	116.67 (8)	C4—C5—H5	120.5 (8)
C2—C1—C6	118.82 (8)	C6—C5—H5	120.4 (8)
C2—C1—H1	120.4 (9)	C5—C6—C1	120.23 (7)
C6—C1—H1	120.8 (9)	C5—C6—C7	119.01 (7)
C1—C2—C3	122.43 (8)	C1—C6—C7	120.75 (7)
C1—C2—Cl1	118.86 (7)	N1—C7—C6	122.74 (8)
C3—C2—Cl1	118.70 (6)	N1—C7—H7	124.9 (10)
C4—C3—C2	117.34 (8)	C6—C7—H7	112.3 (10)
C4—C3—H3	117.8 (11)	N1—C8—C8^i^	109.68 (10)
C2—C3—H3	124.8 (11)	N1—C8—H8A	109.1 (8)
C3—C4—C5	122.12 (8)	C8^i^—C8—H8A	109.6 (8)
C3—C4—Cl2	118.59 (6)	N1—C8—H8B	112.8 (9)
C5—C4—Cl2	119.29 (7)	C8^i^—C8—H8B	109.2 (9)
C4—C5—C6	119.05 (8)	H8A—C8—H8B	106.4 (12)
			
C6—C1—C2—C3	0.08 (14)	C4—C5—C6—C1	0.58 (14)
C6—C1—C2—Cl1	−179.26 (7)	C4—C5—C6—C7	−178.13 (8)
C1—C2—C3—C4	0.42 (14)	C2—C1—C6—C5	−0.59 (14)
Cl1—C2—C3—C4	179.76 (7)	C2—C1—C6—C7	178.10 (8)
C2—C3—C4—C5	−0.43 (14)	C8—N1—C7—C6	179.56 (8)
C2—C3—C4—Cl2	179.97 (7)	C5—C6—C7—N1	−177.99 (9)
C3—C4—C5—C6	−0.06 (14)	C1—C6—C7—N1	3.30 (14)
Cl2—C4—C5—C6	179.54 (7)	C7—N1—C8—C8^i^	−127.06 (11)

Symmetry codes: (i) −*x*+2, −*y*, −*z*+1.

### Hydrogen-bond geometry (Å, °)

**Table:**

*D*—H···*A*	*D*—H	H···*A*	*D*···*A*	*D*—H···*A*
C1—H1···Cl2^ii^	0.962 (14)	2.830 (16)	3.6479 (9)	143.5 (13)

Symmetry codes: (ii) −*x*+1, *y*−1/2, −*z*+1/2.
